# Comparative Efficacy of Face-to-Face and Right-Rear Upright Intubation in a Randomized Crossover Manikin Study

**DOI:** 10.5811/westjem.39983

**Published:** 2025-07-10

**Authors:** Cheng-Wei Tseng, Chung-Shiung Wen, Sheng-Han Yu, Yung-Cheng Su, Shu-Sheng Li, Hsin-Ling Chen, Tzu-Yao Hung

**Affiliations:** * Taipei City Hospital, Zhong-Xing Branch, Department of Emergency Medicine, Taipei City, Taiwan; † Chiayi Christian Hospital, Ditmanson Medical Foundation, Department of Emergency, Chiayi County, Taiwan; ‡ National Yang Ming Chiao Tung University, Faculty of Medicine, Taipei City, Taiwan; § CrazyatLAB (Critical Airway Training Laboratory), Taipei City, Taiwan

## Abstract

**Introduction:**

Upright intubation is essential for managing difficult airways but can be challenging, especially for less experienced clinicians. Face-to-face intubation may lower first-pass success rates due to unfamiliar orientation. New videolaryngoscope devices have the potential to improve intubation success. We aimed to compare first-pass success rates, intubation duration, and glottic view between the right-rear and face-to-face approaches, using channeled videolaryngoscope, hyperangulated videolaryngoscope, and video stylet for upright intubation.

**Methods:**

We conducted a cross-over manikin simulation study involving 30 participants—19 attending physicians, six residents, and five nurse practitioners—to compare the efficacy of these devices to a standard Macintosh videolaryngoscope, using both right-rear and face-to-face approaches.

**Results:**

We used Cox regression analysis to calculate hazard ratios for the following variables: first-pass success rate; intubation time; glottic view quality (Cormack-Lehane grade [C-L]); and percentage of glottis opening score (POGO]. The right-rear approach demonstrated a substantial improvement in first-pass success rates compared to face-to-face, with rates of 93% vs 78% and a hazard ratio of 2.10 (95% confidence interval 1.58–2.80). Additionally, both the video stylet and channeled videolaryngoscope techniques further optimized first-pass success rates and enhanced glottic visualization, achieving a CL grade I view and POGO scores of 100%, even in the inverted face-to-face orientation. These devices outperformed the standard Macintosh and hyperangulated videolaryngoscopes.

**Conclusion:**

The right-rear approach was associated with higher first-pass success rates and provided a more familiar orientation for operators during upright intubation. Video stylets and channeled videolaryngoscopes also contributed to improved success rates, shorter intubation times, and better glottic visualization.

## INTRODUCTION

Compared to the supine intubation position, the upright position for oxygenation and pre-intubation preparation offers several physiological advantages, especially in patients with respiratory compromise or those at risk of hypoxia.^1^,^2^ It reduces abdominal pressure on the diaphragm, allowing for greater lung expansion and enhanced diaphragmatic movement. This leads to increased tidal volumes and improved ventilation. Computed tomography has shown that inspiratory and expiratory lung volumes are 5.3–14.7% higher in the upright position when compared to the supine position,^3^ and functional residual capacity increases by approximately 450 milliliters.^4^

The upright posture also improves gas exchange by optimizing the ventilation-perfusion ratio, particularly in the lung bases, where perfusion is greatest.^1^,^2^ In contrast, the supine position may promote ventilation of less-perfused regions, increasing physiological dead space and impairing oxygenation.^4^ These benefits make upright positioning especially valuable during pre-oxygenation and in patients requiring prolonged airway management.

In patients with elevated intra-abdominal pressure (eg, those with obesity, ascites, or in late-stage pregnancy), the upright position reduces diaphragmatic compression, facilitates full lung expansion, and extends the safe apnea time for intubation.^5^,^6^ Additionally, the supine position can lead to upper airway obstruction due to posterior displacement of the tongue and pharyngeal tissues, particularly in sedated or paralyzed patients.^5^ The upright position helps preserve airway patency, improving ventilation and oxygenation in critical settings.^1^,^2^,^5^,^6^ Upright positioning is also preferred in patients with limited neck mobility, such as those with ankylosing spondylitis, cervical spine injury, or prior head and neck radiation therapy, as it reduces the risk of spinal trauma and facilitates safer airway access.^10^–^13^ In confined environments, such as vehicles, tunnels, or disaster scenes, the upright position may be the only viable option for airway management.^14^

In these scenarios, the face-to-face approach is often described in emergency and prehospital literature as a practical method for upright intubation.^9^,^15^ However, despite its reported equivalence to supine intubation in terms of first-pass success and intubation time,^15^ face-to-face can be technically challenging due to its inverted spatial orientation and non-standard hand positioning. The right-rear approach appears to offer a more ergonomic and intuitive alternative, but its effectiveness has not been thoroughly evaluated.

In parallel, the evolution of videolaryngoscopy, including channeled, hyperangulated, and video stylet designs, has significantly impacted airway management strategies.^12^,^17^–^19^ While these devices have become more prevalent, especially post-COVID-19, evidence regarding their performance in upright intubation remains limited.^18^–^20^ Most prior studies have focused on the supine position, leaving a gap in data regarding upright scenarios, particularly when combined with different approach techniques.

Although flexible fiber-optic intubation offers precise control and is advantageous for managing complex airways, its application in emergency and prehospital settings is limited by several practical constraints. First, it requires substantial technical proficiency, including fine motor skills and familiarity with the equipment—skills that necessitate extensive training and may be difficult to acquire or retain in time-critical environments.^21^ Second, the technique depends on a clear and unobstructed airway, which is often compromised in prehospital scenarios due to blood, secretions, or limited lighting and visibility.^22^ Third, its performance in real-world emergencies remains inconsistent; for instance, recent data indicate a 12.7% first-attempt failure rate during emergency fiber-optic intubations, emphasizing the need for more intuitive and broadly accessible alternatives.^23^ These limitations underscore why, despite its theoretical benefits, fiber-optic intubation may not be the most practical first-line method for upright airway management in high-acuity or resource-constrained environments such as emergency departments (ED) or prehospital care.

**Population Health Research Capsule**

What do we already know about this issue?

*Upright intubation offers physiological advantages in respiratory distress. The face-to-face method is technically challenging; videolaryngoscopy may enhance success rates*.

What was the research question?

*Which approach yields a higher success rate for upright intubation: face-to-face or right-rear?*

What was the major finding of the study?

*The right-rear approach had a 93% first-pass success rate vs 78% with face-to-face; hazard ratio 2.10 (95% CI 1.58–2.80, P < .001)*.

How does this improve population health?

*Optimizing upright intubation by using the right-rear approach with a video stylet or channeled videolaryngoscope can improve patient safety and outcomes*.

In this study we aimed to identify the most effective methods and tools for upright intubation using advanced videolaryngoscopes. We compared the right-rear and face-to-face approaches across four devices: the standard geometric Macintosh videolaryngoscope; channeled videolaryngoscope; hyperangulated videolaryngoscope; and video stylet. Primary outcomes included first-pass and overall intubation success rates, intubation time, and glottic view quality. Through this investigation, we sought to inform best practices for upright airway management, especially in challenging clinical and environmental conditions.

## METHODS

This randomized, cross-over manikin trial was approved by the institutional review board on November 28, 2023 (approval no.: TCHIRB-11211008-E), and was funded by the Department of Health, Taipei City Government (grant number: 11401-62-012). A total of 30 participants were recruited from multiple medical centers across Taiwan, including 19 attending physicians, six residents, and five nurse practitioners (NP). The participants had an average of 11.9 ± 6.43 years of clinical experience in either emergency medicine or anesthesiology. All participants had performed over 100 intubations annually and had at least two years of clinical practice in their respective specialties. To minimize bias, none had prior experience with upright face-to-face or right-rear intubation, which could have otherwise conferred a potential advantage.

**Figure 1:**
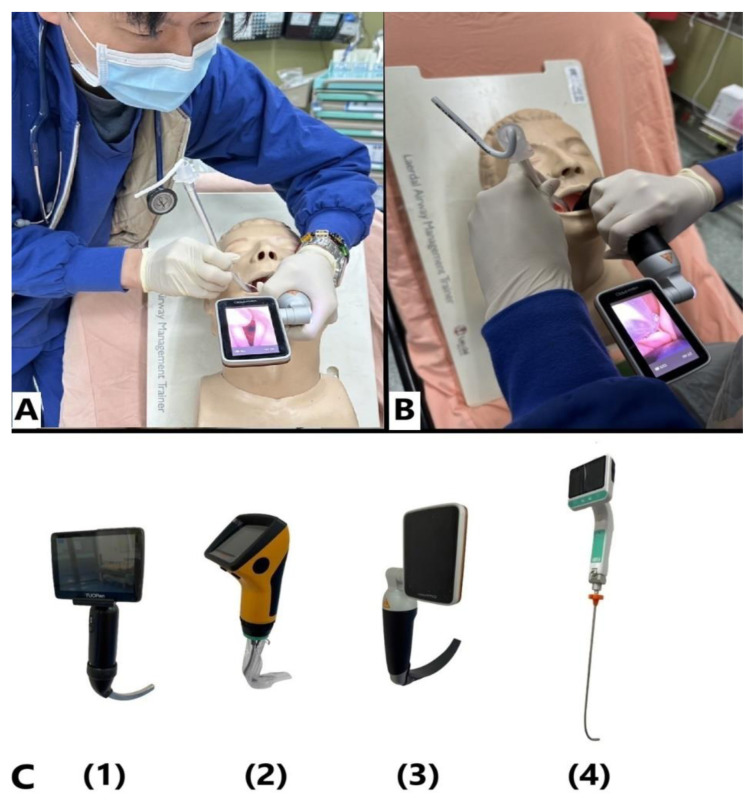
A. Right rear approach: Participants stood at the upper right, posterior side of the patient during intubation. Using their left hand to control the videolaryngoscope and their right hand to pass the tube, they maintained a consistent spatial orientation. B. Face-to-face approach: Participants used their right hand to push the tongue to the right, creating a clear path for tube insertion with their left hand. This approach involved an inverted orientation. C. Device labels: Standard geometric Macintosh videolaryngoscopeChanneled videolaryngoscopeHyperangulated videolaryngoscopeVideo stylet

Prior to the study, all participants underwent structured training in both intubation approaches (Figure 1A and 1B) using each of the four devices (Figure 1C), resulting in eight total approach-device combinations. Participants were required to achieve a minimum of three successful intubations per combination to ensure procedural proficiency and readiness for study participation. Additionally, the chart abstractors were blinded to the study hypothesis to prevent bias during data collection.

### Protocol

Four intubation devices were used: the standard geometric Macintosh videolaryngoscope (Touren Corporation, Gurgaon, India), channeled videolaryngoscope (ITL-SL, AWS-S200, Pentax Corporation, Tokyo, Japan), hyper-angulated videolaryngoscope (HyMac 3, VisionPRO, HEINE Optotechnik GmbH & Co. KG, Germany), and videostylet (TVI-4102, Trachway, Grandmedical Enterprise LTD., Taichung, Taiwan). Each device was tested with two approaches—right-rear (Figure 1A) and face-to-face (Figure 1B)—resulting in eight different approach-device combinations. A conventional 7.0-millimeter internal diameter tracheal tube (Covidien, Mallinckrodt Pharmaceuticals Ltd., Surrey, United Kingdom) was used for intubation without additional assistance, with the manikin (Laerdal Airway Management Trainer, Stavanger, Norway) positioned at a 45-degree upright angle. All procedures were recorded by video clips and reviewed.

Using random.org (https://www.random.org/lists/), we randomized each participant’s sequence of eight approach-device combinations before the study. We defined successful intubation as the passage of the tube through the vocal cords within 90 seconds. Intubation time was measured from insertion of the laryngoscope into the manikin’s mouth until the tracheal tube passed the vocal cord marker and reached a depth beyond 20 centimeters at the level of the incisors. We retrospectively analyzed additional metrics, including time to obtain a proper glottic view, and overall intubation success or failure, through video review.

### Measurements

We recorded participants’ years of experience in the hospital and their specialties (attending physicians, residents, or NPs). The primary outcome measured was the first-pass intubation rate across the eight subgroups, while secondary outcomes included the overall success rate and the total intubation duration.

### Data Analysis

We performed a sample size calculation using the chi-square test for two independent proportions. This calculation was based on successful intubation rates of 78% and 42%, as observed in the standard Macintosh videolaryngoscope group in a previous study involving various intubation scenarios.^16^ To achieve 80% statistical power at an alpha level of 0.05, a minimum of 28 participants per group was required. To account for potential variability due to repeated measures within the same participants, we recruited a total of 30 participants per group.

The primary outcomes of this study were the first-pass success rate. We also analyzed participants’ characteristics, glottic views during intubation (using the Cormack-Lehane classification and the percentage of glottic opening score), overall success rate, and intubation times. The intubation times were divided into 1) the duration from start to glottis visualization, and 2) the duration from glottis visualization to intubation completion.

To evaluate the time to successful intubation, we plotted Kaplan-Meier survival curves to identify trends. To account for correlated data arising from multiple intubation attempts by the same participants, we applied Cox proportional hazards regression models with stratification. These models estimated hazard ratios (HR) for successful intubation, adjusting for potential confounders such as participants’ years of experience, professional roles, intubation devices used, and approach positions.

We conducted data analyses using SAS v9.4 (SAS Institute Inc., Cary, NC) and STATA v17 (StataCorp, College Station, TX).

## RESULTS

The 30 participants included 19 attending physicians, six residents, and five NPs (Table 1). The first-pass success rate for all intubation attempts was 85%. The first-pass success rate for the right-rear approach was 93%, compared to a lower rate of 78% for the face-to-face approach, with all intubations successful within 90 seconds (Table 2). The median times to achieve successful intubation were 10 seconds for the right-rear approach and 13 seconds for the face-to-face approach. Within the right-rear approach, the first-pass success rate was 93% using the standard Macintosh device, 100% with the channeled videolaryngoscope, 77% with the hyperangulated videolaryngoscope, and 100% with the video stylet. Conversely, under the face-to-face approach, the first-pass success rate was 67% with the standard Macintosh videolaryngoscope, 100% with the channeled video laryngoscope, 47% with the hyperangulated videolaryngoscope, and 100% with the video stylet (Table 2). The median intubation times varied with the device and approach. For the right-rear approach, the standard Macintosh device was 10.5 seconds; the channeled videolaryngoscope 9 seconds; the hyperangulated videolaryngoscope 10.5 seconds, and the video stylet 7 seconds; for the face-to-face approach, the standard Macintosh device took 16 seconds, the channeled videolaryngoscope 11 seconds, the hyperangulated videolaryngoscope 26.5 seconds, and the video stylet 11 seconds

**Figure 2:**
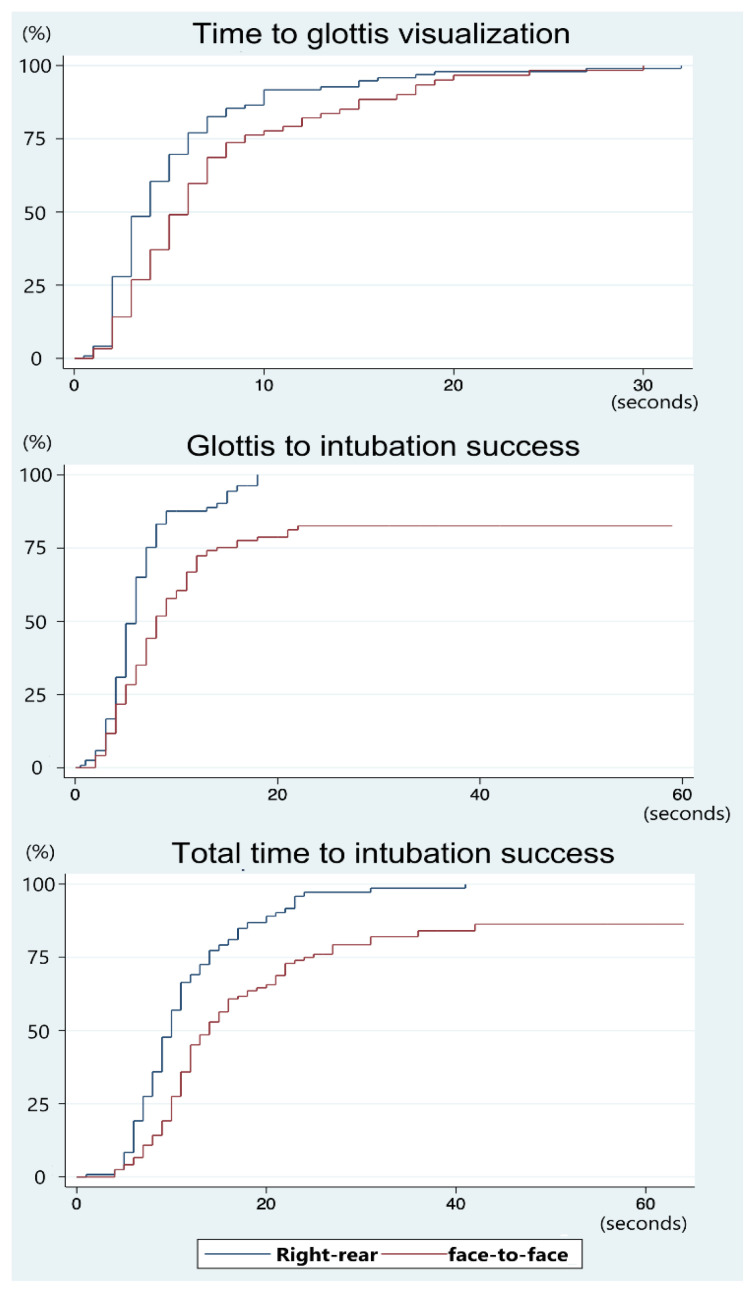
Kaplan-Meier failure estimates for first-pass intubation success: comparison of right-rear vs face-to-face approaches.

**Figure 3:**
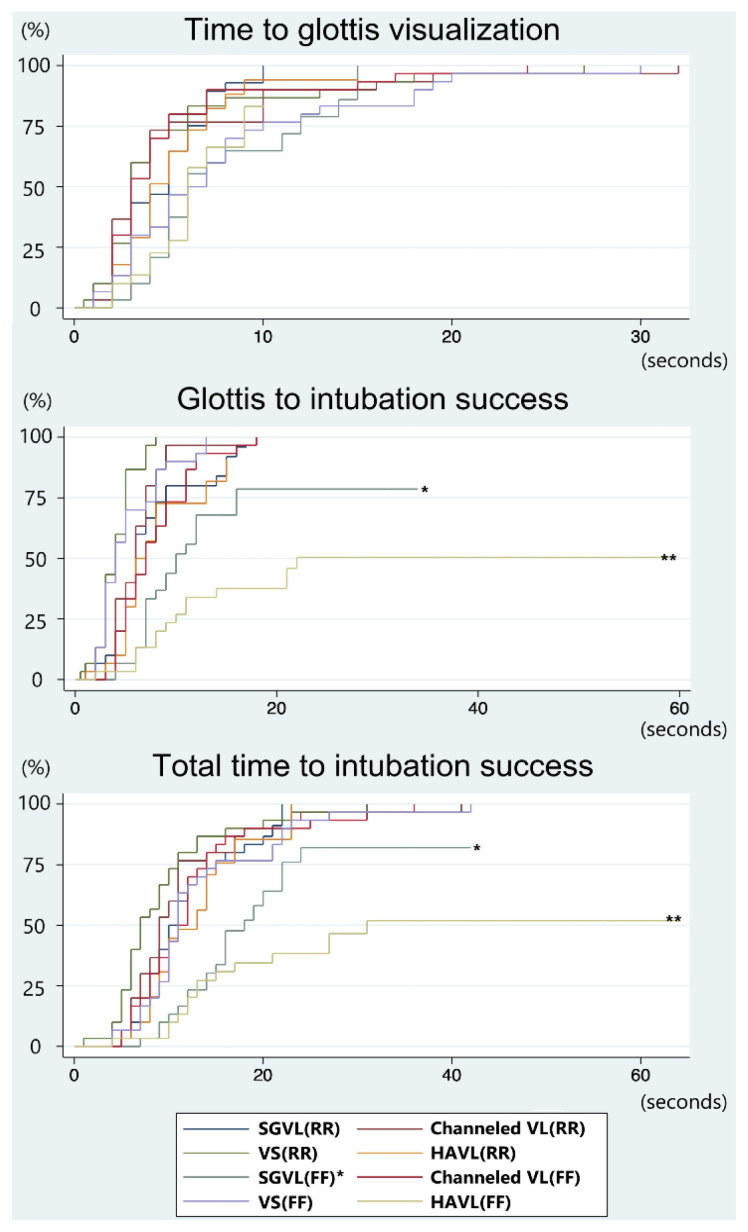
Kaplan-Meier failure estimates for first-pass intubation success across four devices and two approaches, totaling eight subgroups. A)=. Time from the start of intubation to glottic visualization. B. Time from glottic visualization to successful intubation. C. Total duration from the start of intubation to success The face-to-face approach with SGVL(*) and HAVL(**) devices showed lower success rates and longer times for tube passage and overall intubation success compared to other methods. *RR*, right-rear approach; *FF*, face-to-face approach; *SGVL*, standard geometric videolaryngoscope; *VL*, channeled videolaryngoscope; *HAVL*, hyperangulated videolaryngoscope; *VS*, video stylet.

Figure 2 presents a Kaplan-Meier plot comparing the first-pass success rates over time for two intubation approaches: right-rear and face-to-face. The right-rear approach demonstrates an earlier visualization of the glottis compared to the face-to-face approach. Once the glottis was visualized, most intubations in the right-rear group were successfully completed within 20 seconds. The median time to glottic visualization was three seconds for the right-rear approach and five seconds for the face-to-face approach (Table 2). In contrast, the face-to-face approach not only required more time to complete intubation but also demonstrated a lower overall first-pass success rate. Figure 3 further compares the time from glottic visualization to successful intubation across four different intubation devices. Notably, the face-to-face approach is associated with significantly longer times when using the standard Macintosh device and the hyperangulated videolaryngoscope devices compared to the right-rear approach.

In the multivariate Cox regression analysis, no significant effects were found for participants’ age, duration of service, device order, or tenure (Table 3). However, the right-rear approach showed a significant effect, with a HR of 2.10 compared to the face-to-face approach, achieving a *P*-value of <.001 and 95% confidence intervals (CI) ranging from 1.58–2.80. When compared to the standard geometric videolaryngoscope, the channeled videolaryngoscope had a HR of 1.61 (*P*=.02). The hyperangulated videolaryngoscope had a HR of 0.62 (*P*=.03), and for the video stylet, the HR was 1.88 (*P*=.001). There was no significant difference in intubation success between attending physicians, residents, and NPs (Table 3). However, when using the face-to-face approach, attending physicians achieved a better glottic view compared to residents and NPs, with a Cormack-Lehane grade I vs grade IIa, respectively (Table 2).

## DISCUSSION

This randomized crossover simulation manikin study evaluated two upright intubation techniques—right-rear and face-to-face—using four videolaryngoscopic devices. Among experienced clinicians, the right-rear approach yielded significantly higher first-pass success rates (93% vs 78%), faster intubation times, and superior glottic visualization, with a HR of 2.10 (95% CI 1.58–2.80, *P* < .001). The right-rear approach also consistently achieved favorable Cormack-Lehane grade I views and 100% percentage of glottic opening scores, compared to grade IIa and 90% percentage of glottic opening score with the face-to-face approach (Table 2). These findings suggest that the right-rear approach provides a more ergonomically intuitive alignment, resembling conventional midline intubation, which may ease hand-eye coordination and reduce the technical challenges associated with the inverted face-to-face orientation.

Importantly, device selection also played a critical role in performance. Both the video stylet and channeled videolaryngoscope achieved a 100% first-pass success rate across both approaches. Their consistent efficacy, even in the technically demanding face-to-face position, highlights their value in optimizing upright intubation. These devices reduce reliance on precise tube manipulation and glottic angle alignment, making them especially advantageous in scenarios where orientation or operator experience may be limited. Taken together, our results underscore that both approach familiarity and device design must be considered when planning upright airway management strategies.

Although the overall first-pass success rate of 85% in this study may be considered low, it reflects the technical challenges of upright intubation, particularly with the less familiar face-to-face approach. In contrast, the right-rear approach achieved a higher first-pass success rate of 93%, underscoring the importance of spatial orientation and ergonomic familiarity. The lack of significant differences in first-pass success rate among attending physicians, residents, and NPs may be attributed to their uniformly high experience levels and the standardized pre-study training. However, attending physicians demonstrated superior glottic views in face-to-face scenarios, suggesting subtle performance differences not fully captured by first-pass success rate alone (Tables 1 and 2).

Turner et al investigated the feasibility of intubating in an upright position in the ED, reporting that the first-pass success rate increased with every 5° increment in patient positioning, reaching its highest at ≥45 degrees. This challenges the traditional preference for the supine position and aligns with other observational studies suggesting that an upright position may enhance intubation success.^7^,^9^ While face-to-face intubation is widely used in upright scenarios and has been reported as either superior or non-inferior to the standard midline approach,^9^,^15^ we found that the right-rear approach significantly outperformed the face-to-face approach, with the same HR of 2.10 (95% CI 1.58–2.80, *P* < 0.001) (Table 3).

Additionally, the right-rear approach produced better glottic views, achieving Cormack-Lehane grade I and a percentage of glottic opening score of 100%, compared to Cormack-Lehane grade IIa and 90% percentage of glottic opening with the face-to-face approach (Table 2). This difference may be attributed to the more familiar operator orientation in the right-rear position. Moreover, the right-rear approach uses the left hand for laryngoscope manipulation, aligning with standard practice, whereas the face-to-face approach requires the right hand in a mirrored position, which may hinder fine laryngoscope-tip control. Intubation in the face-to-face approach may also be delayed due to tube passage with the non-dominant left hand (Figure 2).

Regarding device performance, the hyperangulated videolaryngoscope has been reported to perform faster than other devices and has been proposed as an alternative to flexible fiber-optic laryngoscopy in awake, upright patients.^19^ Similarly, channeled videolaryngoscope and video stylet have demonstrated greater efficiency than the standard Macintosh device in managing anatomically difficult airways.^16^ Julliard et al. found no significant difference in first-pass success rates between upright and supine use of the hyperangulated videolaryngoscope in cadaver models.^15^ However, in our study, the hyperangulated videolaryngoscope demonstrated the lowest first-pass success rate among the devices tested—even lower than the standard Macintosh device. Specifically, the hyperangulated videolaryngoscope showed a HR of 0.62 (95% CI 0.40–0.96, *P* = .03) (Table 3, Figure 3). Despite participants adapting the stylet angle to match the the hyperangulated videolaryngoscope blade, this adjustment added complexity. The face-to-face approach likely compounded the difficulty due to the required inverted orientation and limited maneuverability, potentially explaining the higher failure rates.

In contrast, the first-pass success rate was highest with the video stylet and channeled videolaryngoscope, outperforming the standard Macintosh device with HRs of 1.88 (95% CI 1.28–2.77, *P* = .001) and 1.61 (95% CI 1.10–2.36, *P* = .02), respectively (Table 3). The video stylet eliminates the need to lift or manipulate the tongue, maintaining consistent performance regardless of spatial orientation. The channeled videolaryngoscope likewise provides a central visual marker and a dedicated tube channel, facilitating efficient tube delivery, even in the inverted face-to-face position. Both devices consistently produced optimal glottic views (Cormack-Lehane grade I, 100% percentage of glottic opening score). In contrast, the standard Macintosh device and the hyperangulated videolaryngoscope had lower first-pass success rates (93% and 77% with the right-rear approach, and 67% and 47% with the face-to-face approach, respectively) and required longer intubation times, especially when used with the face-to-face approach. These differences may be attributed to the increased hand coordination required by the standard Macintosh device and the hyperangulated videolaryngoscope, particularly under non-standard orientations (Table 2, Figure 3).

## LIMITATION

This study did not include induction techniques, which may affect intubation conditions and first-pass success in clinical settings. Our manikin-based simulation allowed for standardized comparisons but lacked patient-specific anatomical and physiological variability. Participants, although experienced in airway management, were more familiar with the head-elevated midline approach (25–30 degrees) than the fully upright (≥45 degrees) right-rear or face-to-face techniques.

The right-rear approach aligned more closely with standard ergonomics, while the face-to-face method involved an unfamiliar, inverted orientation. In practice, face-to-face intubation is rarely used and typically reserved for confined spaces or scenarios where upright positioning is required without prior training. Although simulation drills may improve proficiency, such training is not always feasible. We selected the right-rear approach as a practical alternative to midline intubation for upright scenarios involving cervical spine immobilization or limited neck mobility. Its familiar orientation may make it more broadly applicable in real-world situations.

## CONCLUSION

In this single-center simulation study, the right-rear approach to upright intubation provided a more familiar spatial orientation and was associated with a higher first-pass intubation success rate (93% vs 78%) and better glottic visualization compared to the face-to-face approach, particularly among clinicians without prior experience using the face-to-face technique. The right-rear approach demonstrated a hazard ratio of 2.10 relative to the face-to-face method.

Among the devices tested, video stylets and channeled videolaryngoscopes achieved the highest first-pass success rates and optimal glottic views, even when used in the technically challenging face-to-face orientation. Their design reduced the need for complex hand coordination and improved procedural efficiency. These findings suggest that combining the right-rear approach with either a video stylet or a channeled videolaryngoscope may enhance intubation performance in upright scenarios. However, further clinical studies are needed to validate these results in real-world patient care settings.

## Supplementary Information







## Figures and Tables

**Table 1:** Participant characteristics.

Variable		Mean	Standard deviation
Age (years)		39.33	±6.15
Duration of practice (years)		11.9	±6.43
Total participants	N=30		
Attending physician	19		
Resident	6		
Nurse practitioner	5		

**Table 2:** Study outcomes across two approach methods and four intubation devices.

Variables	N	First-pass success rate	Overall success rate	Time to glottis (s)	Glottis to success (s)	Time to success (s)	POGO score (%)	CL-grade
		Mean±SD	Mean±SD	Median (IQR)	Median (IQR)	Median (IQR)	Median (IQR)	Median
Right-rear approach	120	0.93±0.26	1.00	3 (2–32)	6 (4–7)	10 (7–13)	100 (80–100)	I
Face-to-face approach	120	0.78±0.41	1.00	5 (3–7)	8 (5–12.5)	13 (10–21.5)	90 (60–100)	IIa
Variables of right-rear approach								
Attending physician	76	0.92±0.27	1.00	3 (2–5)	6 (4–7)	9 (7–13)	100 (80–100)	I
Resident	24	0.96±0.20	1.00	5 (2.75–7.25)	5.5 (4–9)	11 (9–16.25)	100 (80–100)	I
Nurse practitioner	20	0.90±0.31	1.00	3 (3–4)	5 (3–6)	9 (6.75–12)	100 (95–100)	I
SGVL	30	0.93±0.25	1.00	4.5 (2–6)	6 (5–8.75)	10.5 (8–14.75)	80 (60–100)	IIa
Channeled VL	30	1.00	1.00	3 (2–4.75)	6 (4–7)	9 (7–11)	100 (100–100)	I
HAVL	30	0.77±0.43	1.00	4 (2.25–5.75)	6 (5–8)	10.5 (9–14)	80 (80–100)	IIa
VS	30	1.00	1.00	3 (2.25–5.75)	4 (3–5)	7 (6–10.75)	100 (100–100)	I
Variables of face-to-face approach								
Attending physician	76	0.78±0.42	1.00	5 (3–7)	8 (5–13)	13 (10.75–22)	100 (60–100)	I
Resident	24	0.92±0.28	1.00	5.5 (4–7.25)	8.5 (6–11.25)	14 (10–20.25)	90 (60–100)	IIa
Nurse practitioner	30	0.65±0.49	1.00	4 (3–5.5)	7 (4–12)	13.5 (10–21.25)	80 (80–100)	IIa
SGVL	30	0.67±0.48	1.00	5 (4–7.75)	9 (7–12.75)	16 (12.5–18.75)	60 (50–80)	IIa
Channeled VL	30	1.00	1.00	3 (2–5)	7 (5–10.5)	11.5 (8–13.75)	100 (100–100)	I
HAVL	30	0.47±0.51	1.00	4.5 (3–6)	20.5 (9.25–31)	26.5 (13–33.75)	60 (60–80)	IIa
VS	30	1.00	1.00	6.5 (3–9.75)	4 (3–7.75)	11 (9.25–14.75)	100 (100–100)	I

*s*, seconds; *POGO*, percentage of glottic opening; *CL*, Cormack-Lehane; *IQR*, interquartile range 25th and 75th percentiles; *SGVL*, standard geometric Macintosh videolaryngoscope; *VL*, channeled videolaryngoscope; *HAVL*, hyperangulated videolaryngoscope; *VS*, video stylet.

**Table 3:** Cox regression analysis of covariate results.

Variable	Hazard ratio	95% Confidence intervals	P-value
Age	1.00	0.93–1.07	0.93
Duration of practice	1.05	0.99–1.12	0.10
Device order	0.98	0.92–1.04	0.42
Approach			
Right-rear vs face-to-face approach	2.10	1.58–2.80	<0.001
Job title			
Nurse practitioner vs attending physician	1.07	0.72–1.58	0.76
Resident vs attending physician	1.53	0.99–1.12	0.13
Device comparison			
HAVL vs SGVL	0.62	0.40–0.96	0.03
Channeled VL vs SGVL	1.61	1.10–2.36	0.02
VS vs SGVL	1.88	1.28–2.77	0.001

*SGVL*, standard geometric videolaryngoscope; *VL*, channeled videolaryngoscope; *HAVL*, hyperangulated videolaryngoscope; *VS*, video stylet.
