# Supplementary figures and images for: Comparative Efficacy of Face-to-Face and Right-Rear Upright Intubation in a Randomized Crossover Manikin Study
Source: West J Emerg Med. 2025 Jul 10;26(4):1086–94. doi: 10.5811/westjem.39983 (PMC12342571; doi:10.5811/westjem.39983)

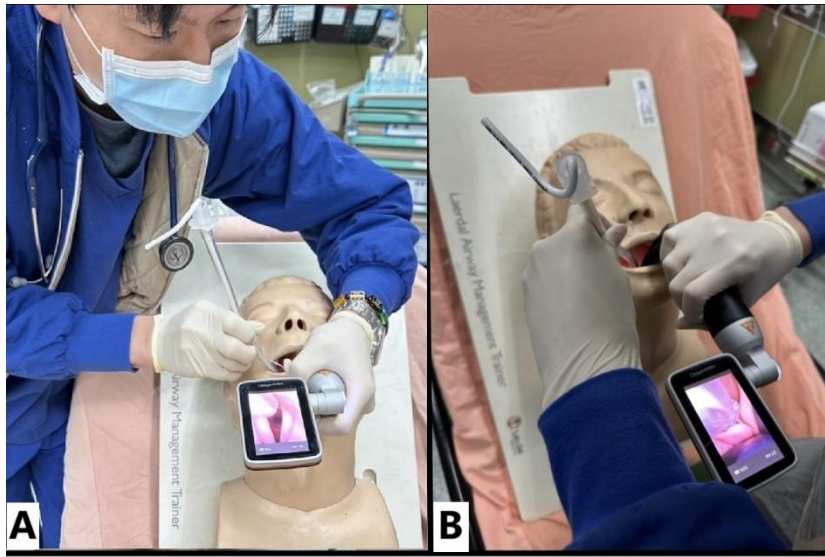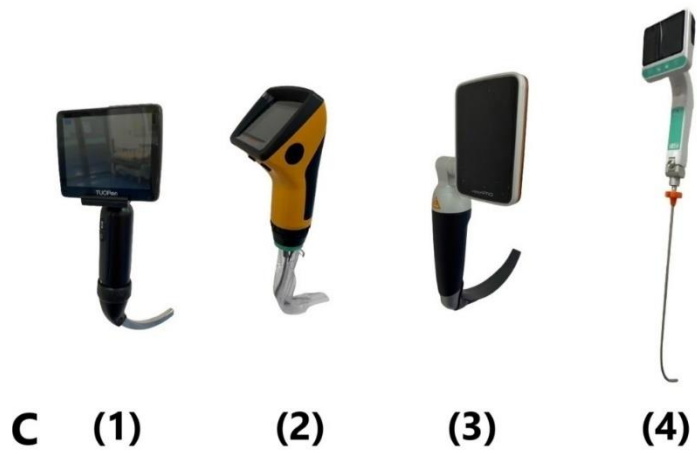

Supplement: Supplementary file 1 [file wjem-26-1086-g001.pdf]

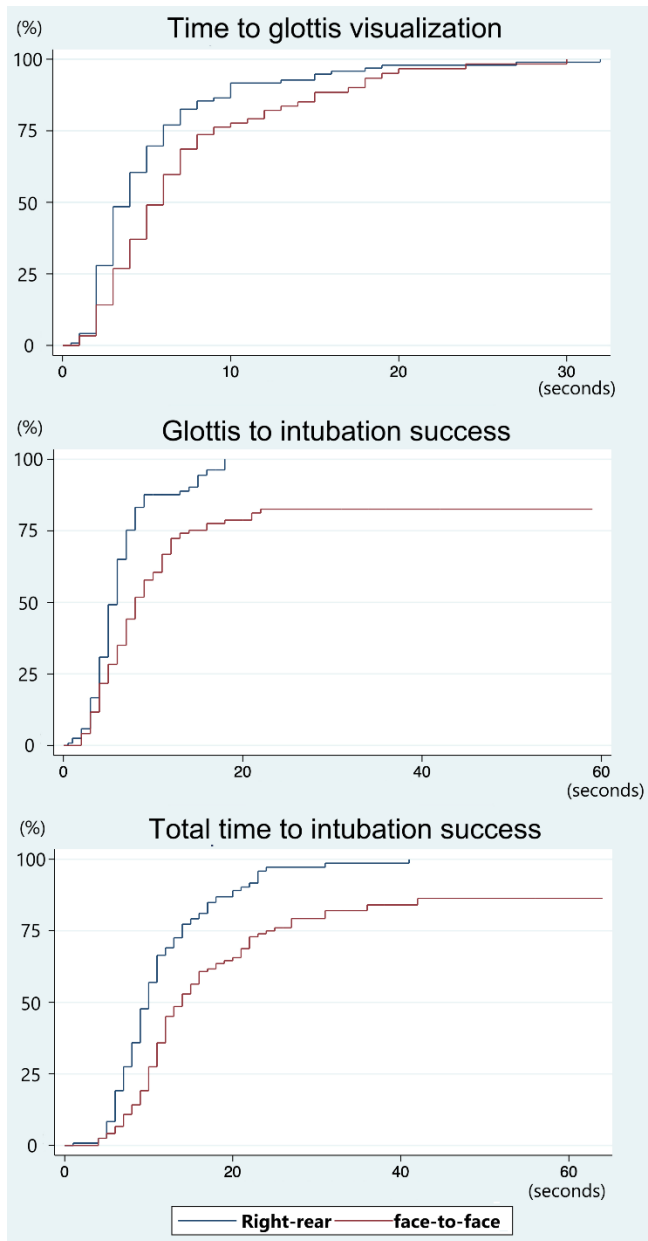

Supplement: Supplementary file 2 [file wjem-26-1086-g002.pdf]

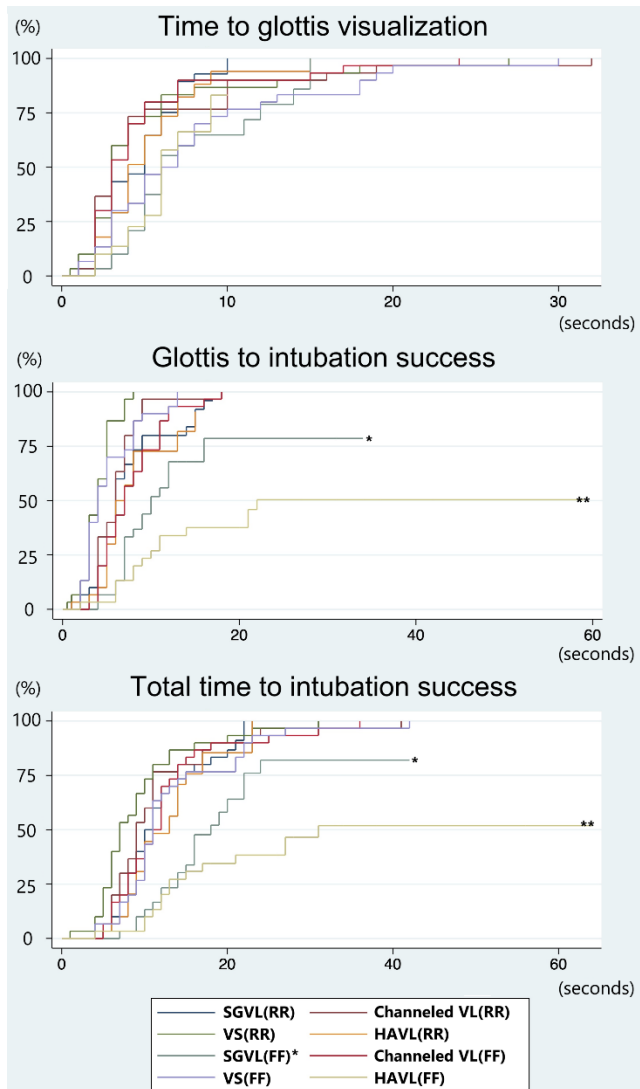

Supplement: Supplementary file 3 [file wjem-26-1086-g003.pdf]
